# Light signaling in plants—a selective history

**DOI:** 10.1093/plphys/kiae110

**Published:** 2024-03-02

**Authors:** Enamul Huq, Chentao Lin, Peter H Quail

**Affiliations:** Department of Molecular Biosciences and The Institute for Cellular and Molecular Biology, The University of Texas at Austin, Austin, TX 78712, USA; Basic Forestry and Plant Proteomics Research Center, Fujian Agriculture and Forestry University, Fuzhou 350002, China; Department of Plant and Microbial Biology, University of California, Berkeley, Berkeley, CA 94720, USA; Plant Gene Expression Center, Agricultural Research Service, US Department of Agriculture, Albany, CA 94710, USA

## Abstract

In addition to providing the radiant energy that drives photosynthesis, sunlight carries signals that enable plants to grow, develop and adapt optimally to the prevailing environment. Here we trace the path of research that has led to our current understanding of the cellular and molecular mechanisms underlying the plant's capacity to perceive and transduce these signals into appropriate growth and developmental responses. Because a fully comprehensive review was not possible, we have restricted our coverage to the phytochrome and cryptochrome classes of photosensory receptors, while recognizing that the phototropin and UV classes also contribute importantly to the full scope of light-signal monitoring by the plant.

Our story begins almost a century ago. The USDA established a research program in Beltsville, Maryland, in the 1920s, with the goal of identifying environmental factors that impact the growth and productivity of crop plants grown by US farmers. Light was soon defined as a major regulator of multiple facets of plant growth and development, including seed germination, seedling development, and flowering, the latter through a process they called photoperiodism. In 1936, plant physiologist Harry Borthwick was recruited to the USDA facility to form a new group focused on using basic research to define mechanisms underlying such light responses. An extensive history of the research initiated by these efforts, up until about 1990, can be found in Linda Sage’s book *Pigment of the Imagination: A History of Phytochrome Research* (Sage 1992).

## Phytochrome

### Brief early history (1940s to early 1960s)

**Figure 1.**
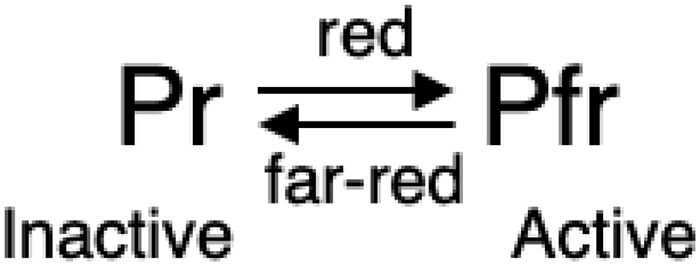
Scheme depicting Hendrick's proposed photoreversible switch-like behavior of the photoreceptor responsible for plant responses to the red/far-red region of the light spectrum. Pr, red-light absorbing form. Pfr, far-red light absorbing form.

In 1940, Sterling Hendricks, an eminent photochemist, joined this effort as a collaborator. He suggested using the strategy of performing action spectra, as this would define the light wavelengths (colors) most active in inducing the relevant plant responses. This work clearly identified the red and far-red wavelengths as the most active in eliciting these responses, thereby defining the light-absorption profile of the molecule in the plant responsible for this activity. A key experiment in 1952 provided critical insight into the unique properties of the receptor molecule (Borthwick et al. 1952). It was found that as little as 1 min of red light was sufficient to trigger germination of lettuce seed but that an immediately subsequent minute of far-red light could cancel the red-light effect, thereby blocking germination. This led Hendricks to conclude that the photoreceptor molecule responsible exists in 2 light-interconvertible forms: a red light–absorbing form (which he called Pr) and a far red–absorbing form (christened Pfr). He also concluded that red light converted Pr to Pfr, far-red light converted Pfr back to Pr, and the Pr form was inactive in inducing seed germination, whereas the Pfr form was active (Fig. 1).

Moreover, he proposed that formation of the Pfr form could be detected physically in the plant by the small increase in far-red absorbance intrinsic to that form upon formation; and conversely, the Pr form would exhibit an increase in red absorbance. Karl Noris, an engineer at the Beltsville facility, was recruited to construct the highly sensitive, specialized spectrophotometer required to detect the extremely small absorbance changes in the plant. Warren Butler, a biophysicist who joined the group in 1956, first succeeded in directly detecting the photoreceptor in dark-grown turnip seedlings in 1959 using Noris's spectrophotometer (Butler et al. 1959). That same day, Harold (Bill) Siegelman, a biochemist who had joined the group in 1957, showed that the activity was also detectable in crude homogenates of the tissue. Butler proposed the name “phytochrome” for the new molecule, and this was officially announced by Borthwick and Hendricks in 1960 (Borthwick and Hendricks 1960).

Siegelman, with the help of Winslow Briggs, went on to provide the first purified preparations of phytochrome, showing that it is a soluble protein with a tetrapyrrole chromophore covalently attached (Siegelman and Firer 1964). This was followed over the next several years by several laboratories that developed additional methods resulting in high-quality preparations of pure phytochrome (Vierstra and Quail 1982) that enabled investigation of the biochemical and photochemical properties of the molecule.

### The early 1960s to late 1970s

As noted by Furuya, little substantive progress toward defining the primary molecular mechanism of phy action in the cell was made during the late 1960s and 1970s (Furuya 1987). This had to wait until the onset of the molecular biology era in the 1980s. One notable exception is the work of Lee Pratt and colleagues starting in the mid-1970s. They produced antibodies to the phy protein and introduced the use of immunochemical procedures, which permitted in vitro analysis of the phy protein (e.g. immunoprecipitation, western blotting) and in situ behavior of the phy molecule at the tissue and subcellular levels (Mackenzie et al. 1975, 1978).

### The early 1980s to the present—photoreceptor molecularly defined

The 1980s saw the dawn of the molecular biology and molecular genetics era in plants. As was the case more broadly, this had enormous impact on the phy field. One early major focus on was to isolate and characterize phytochrome genes from various species, including *Avena sativa*, zucchini, and cucurbita (Hershey et al. 1984). However, *Arabidopsis thaliana* soon became the preferred model system for both genetic and molecular studies. In 1980, Maarten Koornneef pioneered the use of Arabidopsis for obtaining mutants defective in responsiveness to light as a strategy for eventually identifying the photoreceptor molecule responsible (Koornneef et al. 1980). He isolated several long-hypocotyl (*hy*) mutants, some of which would later help achieve this goal. Using a molecular biological approach, Sharrock and Quail described the isolation and characterization of 3 phytochrome genes (*PHYA-PHYC*) from Arabidopsis (Sharrock and Quail 1989), revealing for the first time that there is not 1 but multiple phytochromes in the cell. Two other Arabidopsis phytochrome genes (*PHYD-PHYE*) were later cloned and characterized (Clack et al. 1994). In parallel, mutants in phytochrome genes were isolated for all 5 phytochromes using genetic screens under various conditions. *phyA* mutants were isolated using a 2-step screen, initially selecting long hypocotyl mutants under continuous far-red light and then wild-type–like seedlings under continuous red light from the progeny of the first round [*hy8* for phyA; (Parks and Quail 1993)]. Koornneef's *hy3* mutant yielded *phyB* mutants, and the *hy1* and *hy2* mutants were found to be defective in chromophore biosynthesis (Koornneef et al. 1980). Both *HY3* and *HY8* loci were later cloned, verifying that they encode *PHYB* and *PHYA* genes, respectively (Dehesh et al. 1993; Reed et al. 1993). *phyC* mutants were isolated using a targeted PCR-based screening procedure from various collections of T-DNA insertional mutants and fast neutron deletion libraries (Monte et al. 2003). A *phyD* mutant was isolated by cloning the *PHYD* gene from the WS ecotype, which was later found to have a 14-bp deletion in this ecotype compared with the Col-0 background (Aukerman et al. 1997). Finally, *phyE* mutants were isolated from a 2-step genetic screen for early flowering and elongated rosette internodes in the first round of screen and then selecting mutants displaying attenuated internode elongation and flowering responses under end-of day far-red treatments from the progeny of the first round (Devlin et al. 1998).

The isolation of individual phytochrome mutants helped define the biological roles and photosensory specificity of each phytochrome. Phenotypic characterization showed that although all phytochromes are activated by red light, individual phytochromes have both distinct and overlapping roles in regulating plant development under various light conditions (Li et al. 2011). Strikingly, the phytochrome-deficient quintuple mutant (*phyabcde*) of Arabidopsis showed that phytochromes are dispensable for completion of the Arabidopsis life cycle, although these mutants are severely defective in developmental responses to the prevailing light environment (Strassera et al. 2010; Hua et al. 2013). More recently, in a major paradigm shift, phytochromes have been shown to function not only as a light receptor but also as a temperature sensor in plants (Jung et al. 2016; Legris et al. 2016). These studies highlight the importance of phytochromes as a broad environmental sensor in regulating plant growth and development.

Two major long-term goals in the field have been to solve the structure of the phytochrome molecule and to understand its functional activity at the molecular level. A breakthrough regarding the first goal came with the success of Vierstra and colleagues, initially in solving the crystal structure of the Pr and Pfr forms of the photosensory domains, and later the full-length phytochromes, from bacteria (Wagner et al. 2005; Essen et al. 2008; Ulijasz et al. 2010). Most recently, using cryo-electron microscopy (cryo-EM), these authors have defined the 3D structures of the Pr forms of the full-length Arabidopsis phyA and phyB molecules (Li et al. 2022; Burgie et al. 2023; Zhang et al. 2023). These structures have revealed that both phyA and phyB are asymmetric dimers in their N-terminal photosensory domain, while they have a symmetric dimer configuration in their C-terminal: histidine kinase-related domains (HKRD). One notable difference between the phyA and phyB structures is that, whereas the phyB N-terminal photosensory domain and the HKRD region associate asymmetrically, these associations are absent in phyA. These data suggest that this decoupling between the N- and C-terminal domains in phyA might have functional consequences for Pfr stability and photosensory specificity.

### Molecular signaling mechanism defined

In parallel with this structural research, efforts were ongoing in multiple laboratories toward the second goal of understanding how these photoreceptors function inside the plant cell. Early immunocytochemical studies had led to the proposal that phys are constitutively localized in the cytosol (Mackenzie et al. 1975) and that the activated form communicates with the nucleus through second messengers to control gene expression (Millar et al. 1994). In support of this hypothesis, a few second messengers, including Ca2+, calmodulin, and cGMP, had been shown to impact gene expression and chloroplast development (Bowler et al. 1994). However, in 1996, Sakamoto and Nagatani provided initial evidence that phyB localizes to the nucleus in the Pfr form (Sakamoto and Nagatani 1996). Following this study, all phytochromes were shown to be initially in the cytoplasm in the Pr form and to then translocate rapidly into the nucleus in response to light, with varying kinetics and fluence-rate specificity (Kircher et al. 1999, 2002; Yamaguchi et al. 1999).

**Figure 2.**
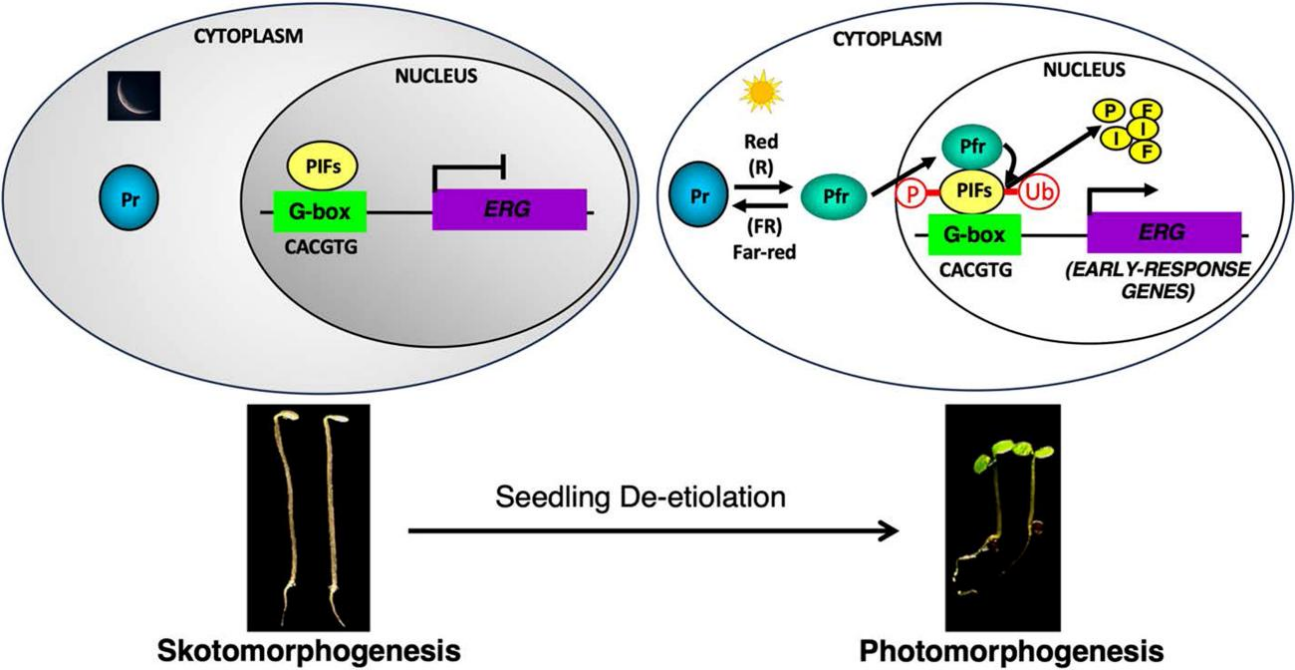
Photoactivated phys directly regulates target-gene expression via molecular interaction with PIF transcription factors. In the dark (left), phytochromes are synthesized in their biologically inactive Pr form, which is localized to the cytosol. PIFs are constitutively nuclear-localized transcription factors that bind to G-box (CACGTG) sequence elements in the promoters of many light-regulated genes. Arabidopsis seedlings undergo etiolated development (skotomorphogenesis). Upon red light illumination (right), phytochromes are converted to the biologically active Pfr form. The Pfr form translocates into the nucleus, binds to PIFs, and induces direct-target gene expression. The phy-PIF interaction within the nucleus results in rapid phosphorylation, ubiquitination, and degradation of PIFs. This light-induced degradation of PIFs results in activation of PIF-repressed genes and suppression of PIF-activated genes. The transcriptional network elicited by light exposure drives photomorphogenic development.

Nagatani's findings converged in the late 1990s with efforts to identify phy-interacting proteins directed toward the goal of defining the molecular mechanism of light-signal transfer in the cell. The breakthrough came when Ni et al. (Ni et al. 1998, 1999) identified a basic helix-loop-helix (bHLH) transcription factor (Toledo-Ortiz et al. 2003), which they called Phytochrome Interacting Factor 3 (PIF3) and which interacted specifically with the Pfr form of phyB (Martinez-Garcia et al. 2000). These findings established that the light-activated photoreceptor molecule signals directly to the genome via physical interaction with components of the transcriptional machinery, thereby regulating the expression of target genes (Fig. 2). It had been noted by Pratt and colleagues in the late 1970s that light-activated phy formed “speckles” (later called “photobodies”) in the nucleus in prolonged irradiation (Mackenzie et al. 1978). In 2004, Schaefer and colleagues showed that light-activated phy rapidly (within minutes) formed photobodies upon nuclear translocation and that constitutively nuclear-localized PIF3 also coalesced together with phy in these photobodies (Bauer et al. 2004). Moreover, they found that this interaction led to rapid degradation of the PIF3 protein.

Using PIF3 as a bait, PIF1 and PIF4, other members of the bHLH family, were also isolated and characterized (Huq and Quail 2002; Huq et al. 2004). When the genome sequence of Arabidopsis became available, a number of additional bHLH PIFs (PIF5-PIF8) were identified and characterized (Khanna et al. 2004, 2007; Leivar et al. 2008a, 2008b; Oh et al. 2020). Among all the PIFs, PIF1 and PIF3 were shown to interact with the Pfr form of phyA and phyB (Huq et al. 2004), whereas all the other PIFs were shown to interact with the Pfr form of phyB. Thus, these transcription factors display differential affinities for the different phys, suggesting the possibility of combinatorial pathway divergence at the genome interface.

In parallel studies, earlier genetic screens to identify mutants in the phy signaling pathways yielded another class of important mutants, named *constitutive photomorphogenic*/*de-etiolated*/*fusca* (*cop/det/fus*) (Chory et al. 1989; Deng et al. 1991). These mutants displayed light-grown phenotypes in darkness, suggesting that these factors act as negative regulators not only of phy signaling but also of other light signaling pathways. Molecular cloning and characterization of the *COP1* gene revealed that this factor is mostly involved in protein degradation through the ubiquitin/26S proteasome pathway (Deng et al. 1992). The COP1 protein functions as an E3 ligase for degradation of positively acting components in light signaling pathways (e.g. ELONGATED HYPOCOTYL 5, HY5 and others) (Osterlund et al. 2000). COP1 has been shown to interact with SUPPRESSOR OF PHYA-105 1 (SPA1-SPA4) family of proteins (Saijo et al. 2003; Seo et al. 2003). The COP1-SPA complex acts as a substrate adaptor for the CULLIN4-based E3 ligase that degrades positively acting transcription factors in light signaling pathways (Xu et al. 2015; Han et al. 2020). Upon photoactivation, phys reverse the COP1-SPA function in promoting photomorphogenesis in 2 ways. On the one hand, light-activated phys interact with the SPA proteins upon translocation into the nucleus, reorganizing the COP1-SPA complex (Lu et al. 2015; Sheerin et al. 2015) and thereby inhibiting the E3 ligase activity. In addition, in prolonged light, COP1 is excluded from the nucleus (Subramanian et al. 2004), thereby reducing the E3 ligase activity within the nucleus. These dual inhibitory mechanisms result in stabilization and accumulation of the positively acting transcription factors (e.g. HY5, LAF1, HFR1, and others), which then promote photomorphogenesis.

To understand the regulatory functions of the PIFs, *pif* mutants were identified, mainly using reverse-genetic approaches, although *pif4* was isolated by both genetic and reverse-genetic approaches (Huq and Quail 2002). As members of the bHLH family, PIFs are highly homologous proteins (Toledo-Ortiz et al. 2003; Castillon et al. 2007; Leivar and Quail 2011). However, phenotypic characterization of the *pif* mutants showed that individual PIFs regulate specific, as well as overlapping, sets of biological pathways. For example, PIF1 and PIF8 regulate seed germination (Oh et al. 2004, 2020); PIF1 and PIF3 regulate chlorophyll and carotenoid biosynthetic pathways (Huq et al. 2004; Stephenson et al. 2009); PIF4, PIF5, and PIF7 regulate shade avoidance responses (Lorrain et al. 2008; Leivar et al. 2012); and all the PIFs regulate hypocotyl lengths to varying degrees under red light (Huq and Quail 2002; Monte et al. 2004; Leivar et al. 2008a, 2008b). Strikingly, higher-order *pif* mutants (e.g. *pif1 pif3 pif4 pif5*, termed *pifQ*) display short hypocotyl and expanded cotyledons in darkness mimicking light-grown phenotypes (Leivar et al. 2008a, 2008b; Shin et al. 2009), suggesting that PIFs function negatively in regulating these processes.

The array of gene expression changes underlying these morphological phenotypes regulated by the individual phys were first defined in the early 2000s using early microarray technology (Tepperman et al. 2001, 2004, 2006). This was followed later by RNAseq studies showing that a large number of light-controlled genes are regulated by the PIFs by comparing the *pifQ* mutant in darkness with the gene expression pattern in the light-exposed wild type seedlings (Leivar et al. 2009; Shin et al. 2009). Moreover, PIFs directly regulated a subset of these genes both individually as well as in an overlapping manner (Zhang et al. 2013; Leivar and Monte 2014; Pfeiffer et al. 2014). Thus, the PIFs function as negative regulators whereas the phys function as positive regulators of photomorphogenesis, establishing a “yin-yang” relationship between the 2 signaling partners.

The earlier discovery that rapid, light-induced colocalization of the phy and PIF3 molecules in photobodies induced rapid degradation of the transcription factor (Bauer et al. 2004) supports the proposal that the promotion of photomorphogenesis by light results from reversal of PIF-imposed repression of this process. Subsequently, a number of studies showed that, with the exception of PIF7 (Leivar et al. 2008a, 2008b), all other PIFs are also degraded in light with varying kinetics and fluence-rate dependency (Park et al. 2004; Shen et al. 2005, 2007; Al-Sady et al. 2006; Lorrain et al. 2008). Among all the PIFs, PIF1 is the most light-sensitive, with a half-life less than 1 min, reflecting its strong interaction with both phyA and phyB (Huq et al. 2004; Shen et al. 2008). Thus, the phy-induced degradation of PIFs has formed the foundation of the primary signaling event in phy signaling pathways. In addition, phyB has been shown to inhibit the DNA binding activity of PIF3 by sequestration (Park et al. 2012). In fact, the N-terminal domain of phyB binds to PIF3 and inhibits the DNA binding and transcriptional activation activity of PIF3, while the C-terminal domain of phyB promotes the degradation of PIF3 (Park et al. 2018; Yoo et al. 2021). This is also consistent with the deetiolated phenotypes of the *pifQ* quadruple mutant in darkness (Leivar et al. 2008a, 2008b), suggesting that the removal of PIFs either genetically or by light-induced degradation or inhibition of PIF function by sequestration is sufficient to promote photomorphogenesis.

Early investigation of the mechanism of phy-induced PIF degradation provided evidence that PIFs are first phosphorylated and then ubiquitinated before being degraded through the 26S proteasome pathway (Fig. 2) (Al-Sady et al. 2006). Intense efforts by various laboratories were then focused on identifying the kinases and E3 ubiquitin ligases for PIFs. In 2014, Ni et al. showed that LRB proteins [Light-Response Bric-a-Brack/Tramtrack/Broad (BTB)] act as substrate adaptors for PIF3 degradation under light (Ni et al. 2014). Moreover, LRBs also act as substrate adaptors for phyB, ensuring mutual co-degradation of the phyB-PIF3 complex in the light. In 2015, Zhu et al. described a different E3 ligase for light-induced degradation of PIF1 (Zhu et al. 2015). These authors showed that COP1-SPA proteins, well-known repressors of photomorphogenesis, act as a substrate adaptor complex in a CUL4-based E3 ligase to recruit PIF1 and PIF5 for light-induced degradation (Zhu et al. 2015; Pham et al. 2018). In addition, Dong et al. showed that EIN3-BINDING F BOX PROTEIN 1 (EBF1) and EBF2, which form the CUL1^EBF1^ and CUL1^EBF2^ complexes, respectively, also recruited PIF3 for degradation (Dong et al. 2017). Moreover, a number of other E3 ligases, including COLD TEMPERATURE-GERMINATING 10 (CTG10) (Majee et al. 2018), BLADE-ON-PETIOLE (BOP) (Zhang et al. 2017), and HIGH EXPRESSION OF OSMOTICALLY RESPONSIVE GENES1 (HOS1), were described to recruit various PIFs for light-induced degradation (Kim et al. 2017a, 2017b). Thus, it appears that multiple E3 ligases can recruit PIFs for light-induced degradation to promote photomorphogenesis.

Following the discovery of E3 ligases, progress in identifying kinases for the relevant PIFs was made by various laboratories. The first candidate was phys themselves, as early evidence showed the C-terminal domain of phys displays sequence homology to the histidine kinase families (Quail 1997), and biochemical evidence suggested that phys might function as a serine threonine kinase (Yeh and Lagarias 1998; Shin et al. 2016). However, recent cryo-EM structures did not reveal any kinase-related domain in either phyA and phyB (Li et al. 2022; Burgie et al. 2023; Zhang et al. 2023). Although the phy kinase hypothesis has remained a hot topic for debate in the field, various laboratories were focused on identifying other kinases for PIFs. The first of these kinases is called the Photoregulatory Protein Kinases (PPK1–PPK4), which were previously known as MUT9-Like Kinases (MLKs), that phosphorylate PIF3 in vitro (Ni et al. 2017). Interestingly, like LRBs, PPKs also controlled the degradation of the phyB-PIF3 complex resulting in a hypersensitive phenotype of the higher order *ppk* mutants. Paik et al. showed that SPA1, previously known as an adaptor for COP1, functions as a kinase for PIF1 (Paik et al. 2019). SPA proteins have a serine threonine kinase related domain at the N terminus (Hoecker et al. 1999), but the functional significance of this domain is unknown. A mutant form of SPA1 with a mutation at the kinase domain failed to rescue the *spaQ* phenotypes, whereas the WT SPA1 largely rescued the phenotypes, suggesting that this domain is important for SPA function. Apart from PPKs and SPAs, a few other kinases have been described for PIFs. These include Casein Kinase 2 (CK2) (Bu et al. 2011) (Bernardo-García et al. 2014) and mitogen- activated protein kinase 6 (MPK6) (Xin et al. 2018a). However, these kinases may not be involved in the light-induced phosphorylation of the PIFs.

In addition to the phy-PIF module-controlled transcriptional regulation of gene expression described above, phys have been shown to control the post-transcriptional and translational steps in gene regulation (Cheng et al. 2021; Kathare and Huq 2021). In 2014, Shikata and colleagues first showed that phys extensively regulate alternative splicing of pre-mRNAs, where variable splice sites were used in multi-intron pre-mRNAs to generate multiple forms of mRNAs from the same transcript (Shikata et al. 2014). In another study, overaccumulation of phyB, especially in the *lrb* mutant backgrounds, was shown to promote intron retention in the 5′ untranslated region of PIF3 mRNA, resulting in inhibition of PIF3 translation (Dong et al. 2017). Following these studies, a number of splicing factors have been identified by genetic and biochemical approaches in both Arabidopsis (Splicing Factor for Phytochrome Signaling, SFPS; Reduced Red-light Responses in Cry1Cry2 background, RRC1, Suppressor of White Apricot 1, SWAP1, and SMP2) (Xin et al. 2018b; Xin et al. 2019; Kathare et al. 2022; Yan et al. 2022) and *Physcomitrella patens* (heterogeneous nuclear ribonucleoproteins, PphnRNP-H1; PphnRNP-F1) (Shih et al. 2019; Lin et al. 2020). Strikingly, all of these splicing factors directly interact with phys. However, unlike PIFs, these splicing factors are not degraded under light, suggesting a distinct mechanism to control their function by phys.

Light signals not only control transcription and post-transcriptional processing of mRNAs but also translation of many mRNAs. The first direct evidence that phys are involved in this process came from identifying a phy-interacting factor named PENTA1 (PNT1) (Paik et al. 2012). PNT1 directly binds to the 5′ untranslated region of the protochlorophyllide reductase A (PORA) mRNA. Light-activated phys interact with PNT1 and repress translation of PORA. In addition, COP1 has been shown to repress translation in darkness (Chen et al. 2018). Light-induced inactivation of COP1 through phys and crys enhances translation of many mRNAs.

Recent findings have unveiled an exciting new direction in defining the functional activities of the phy-PIF duo in the nucleus. The discovery by Chentao Lin and colleagues [(Wang et al. 2021); see below] that light-induced CRY2 photobody formation in the nucleus results from the rapid coalescence of the CRY2 molecules into liquid-liquid droplets highlighted existing long-standing evidence of physical interaction between, and co-occupation of, photobodies by light-activated CRY2 and phys (Yamaguchi et al. 1999; Más et al. 2000), thereby predicting that phy photobody formation likewise results from liquid-liquid phase separation of the photoreceptor molecule (Quail 2021). Direct evidence that light-activated phy is induced to form liquid-liquid phase separated (LLPS) photobodies, that function as signaling hubs of the phys, soon followed (Chen et al. 2022). In parallel, Chory and colleagues have provided elegant evidence of 1 major signaling-hub function of phy-PIF7–harboring photobodies (Willige et al. 2021). These authors show that light-induced, Pfr-activated phosphorylation of PIF7 sequesters the transcription factor in photobodies away from access to its DNA binding sites. Conversely, vegetative shade-induced reduction in Pfr levels results in PIF7 dephosphorylation, releasing it from the photobodies to bind to its target cis-acting DNA sites. The bound PIF7 then ejects the H2A.Z histone variant from chromatin, H3K9 is acetylated, and gene expression of PIF7 target-gene expression is initiated (Willige et al. 2021). Because more than 100 transcription factor genes are directly targeted, an extensive, diverse PIF7-initiated gene network is modulated by this regulatory module.

Kim et al. (2021) have provided additional evidence that phys regulate chromatin remodeling directly and indirectly to control the expression of target genes that promote plant development. They showed that phyB directly interacts with VERNALIZATION INSENSITIVE 3-LIKE1 (VIL1), a component of the Polycomb Repressive Complex 2 (PRC2), in a light-dependent manner (Kim et al. 2021). phyB-VIL1 synergistically represses the expression of growth promoting genes (e.g. ATHB2) through the formation of a chromatin loop in a light-dependent manner that promotes the enrichment of Histone H3 Lys27 trimethylation (H3K27me3), a repressive histone modification. In addition, Gonzalez-Grandio and colleagues have examined whether the rapid activation and repression of PIF direct-target genes are associated with chromatin changes (González-Grandío et al. 2022). These authors found evidence that the light-regulated transcriptional changes and chromatin-remodeling processes might be mechanistically intertwined.

## Cryptochromes

### A brief early history

Blue and red light are the 2 major spectral regions of sunlight used by plants for photosynthesis, and these 2 regions are also the 2 major wavelengths used for photomorphogenesis. In his book “The Power of Movement in Plants,” Charles Darwin described an experiment in his study of the circumnutational movement of cabbage seedlings. In this experiment, “the plants were illuminated by light passing through a solution of bichromate of potassium so as to eliminate heliotropism” (Darwin 1881). Given that heliotropism is primarily a phototropic response and that orange-colored potassium bichromate solution has an absorption spectrum of approximately 310 to 450 nm (from UV-A to blue light) (von Halban et al. 1927), the correlation between removal of UV-A and blue-light photons and abolition of plant heliotropism implied that phototropism is a blue-light response. Indeed, Julius von Sachs, who is considered the father of plant physiology (Kutschera and Niklas 2018), measured the first crude action spectrum of plant phototropic responses and found that it is maximal in the blue-light region (Sachs 1887).

Since the discovery of phototropism, plant biologists have made enormous efforts to identify the molecular mechanism underlying this fascinating blue-light response of plants (Butler et al. 1959; Briggs et al. 1983). For example, Winslow Briggs and his students discovered light-induced auxin transport and demonstrated that this transport was lateral within the plant organ in response to the light, causing asymmetric cell elongation, which led in turn to phototropism (Briggs et al. 1957). However, early efforts to biochemically isolate blue-light receptors encountered many technical difficulties (Briggs et al. 1983), such that molecular identification of blue-light receptors had to wait until 1990s (Briggs and Huala 1999). Because no biochemical activity was known for these receptors at the time, the lack of an appropriate readout imposed a technical hurdle difficult to overcome in those days. The biochemical study of plant blue-light receptors was greatly encouraged by a seemingly promising readout called LIAC (light-induced absorption changes), which was originally discovered in the study of photosynthesis (Kok 1957). It was found that extracts of membrane fractions of fungi, algae, and higher plants exhibited LIAC with an action spectrum similar to that of phototropism, as well as photoreduction of flavoproteins, especially the b-type cytochromes (Poff and Butler 1974; Brain et al. 1977; Briggs et al. 1983). Unfortunately, this seemingly promising approach was also fruitless in identifying the molecular nature of plant blue-light sensory receptors, due to the relatively low abundance of these photoreceptors in comparison to the photosynthetic pigments. Although it was correctly predicted from these earlier studies that the blue-light sensory receptors would be flavoproteins that contain a chromophore of either FAD (Flavin Adenine Dinucleotide) or FMN (Flavin Mononucleotide), none of the presently known plant receptors is a cytochrome and none appear to be responsible for the LIAC phenomenon (Briggs 2010). Despite this setback, Jonathan Gressel, inspired by the successes in identifying the phytochromes, proposed the term “cryptochrome” in 1979 for the-yet-to-be molecularly identified pigments having both the absorption and action spectra in the UV-A/blue light region of the spectrum and actually molecularly responsible for the plant photoresponse (Gressel 1979; Senger 1984). The name cryptochrome was coined “because of importance in cryptogamic plants (in the study of blue-light responses) and its cryptic nature” (Gressel 1979).

### Molecular identification of cryptochromes

Fourteen years later, Cashmore and colleagues molecularly identified the first bona fide blue-light sensory photoreceptor (Ahmad and Cashmore 1993; Cashmore et al. 1999). This achievement was made possible by the molecular-genetic methodologies developed in the model plant *Arabidopsis thaliana* (Koornneef et al. 1980; Feldmann 1991; Somerville and Koornneef 2002). In 1980, in addition to the phytochrome mutants described above in the previous section, Maarten Koornneef also isolated the *hy4* mutant that exhibits a long-hypocotyl phenotype in blue light (Koornneef et al. 1980). In 1991, Kenneth Feldmann generated 8,000 Arabidopsis T-DNA insertion-mutant lines, making it much easier to identify genes corresponding to mutants that have visible phenotypes (Feldmann 1991. In 1993, Anthony Cashmore's laboratory screened this mutant population, identified a T-DNA tagged allele of the *hy4* mutant, *hy4-2*, and cloned the *HY4* gene (Ahmad and Cashmore 1993). The *HY4* gene encodes a protein with an N-terminal domain that is approximately 30% identical to DNA photolyases. Interestingly, a *Sinapis alba* photolyase-like gene (SA-Phr1) was reported (Batschauer 1993) a couple of months before the publication of the *HY4* gene. Because DNA photolyase is a blue light-dependent flavoenzyme that repairs cyclobutane pyrimidine dimers of UV-damaged DNA (Malhotra et al. 1992), these results immediately suggested that the HY4 protein is most likely a blue-light receptor responsible for regulating seedling hypocotyl growth (Ahmad and Cashmore 1993). Consistent with this prediction, both the full-length HY4 protein expressed in and purified from insect cells and the N-terminal domain expressed and purified from *E. coli* as the MBP (Maltose Binding Protein) fusion protein were found to contain oxidized FAD that absorbs UV-A and blue light (Lin et al. 1995; Malhotra et al. 1995). It was observed that the HY4 protein is not only required for a blue light-specific response (Koornneef et al. 1980; Ahmad and Cashmore 1993; Ahmad et al. 1995) but also itself absorbs blue light, fulfilling the 2 key criteria of a blue-light sensory receptor or a cryptochrome; it was named cryptochrome 1, or CRY1 (Lin et al. 1995).

The Arabidopsis genome also encodes a CRY1 homolog that was called CRY2 or PHH1 (Hoffman et al. 1996; Lin et al. 1996). CRY1 and CRY2 share 58% sequence identity in their N-terminal photolyase-like domains and 13% in their C-terminal nonphotolyase domains. Antibodies against CRY2 were prepared and used in a genetic screen to isolate the loss-of-function *cry2* mutants (Guo et al. 1998; Lin et al. 1998). In this experiment, a fast neutron-mutagenized population of M2 seedlings was grown in blue light to select those that grew slightly taller than the wild-type because it was speculated that CRY2 may have overlapping function with CRY1 in mediating blue light inhibition of hypocotyl elongation. The isolates from the first screen were analyzed by immunoblots probed with the anti-CRY2 antibody. This 2-step genetic screen resulted in 2 alleles of the *cry2* mutants that suffer from complete or partial deletion of the *CRY2* gene and absence of the CRY2 protein (Guo et al. 1998; Lin et al. 1998). The *cry2* mutants exhibited 2 abnormal phenotypes. First, the mutant exhibited the long-hypocotyl phenotype compared with wild-type, especially when grown in low intensities of blue light (Lin et al. 1998). Second, this mutant exhibited a later-flowering phenotype compared with the wild type when they were grown in long-day photoperiods or continuous white light (Guo et al. 1998). A screen of the late-flowering mutants known at that time demonstrated that *cry2* is allelic to *fha*, which is one of the many late-flowering mutants previously isolated by Maarten Koornneef (Koornneef et al. 1991). The late-flowering phenotype of the *cry2* mutant is wavelength specific but in a manner more complex than expected. It was known that blue light promotes and red light inhibits flowering in Arabidopsis (Brown and Klein 1971; Eskins 1992). Surprisingly, the *cry2* mutant showed normal flowering time when grown in monochromatic blue light. It turned out that cry2 displayed the late-flowering phenotype only in the presence of both blue light and red light (Guo et al. 1998). Based on this observation, it was proposed that CRY2 mediates blue light inhibition of phyB-dependent suppression of floral initiation (Guo et al. 1998). Indeed, it was demonstrated that phyB mediates red light inhibition of proteolysis of the flowering promoter protein CO (CONSTANS), whereas CRY2 mediates blue light suppression of phyB function and thereby of CO degradation. These experiments explained the puzzling wavelength-specific phenotype of the *cry2* mutant and demonstrated the close functional association of the blue light–receptor cryptochromes and the red light–receptor phytochromes.

Soon after discovery of the Arabidopsis CRY1 and CRY2, the presence of cryptochromes was also reported in animals. It was found that *Drosophila* dCRY acts as the photoreceptor mediating light regulation of the circadian clock in *Drosophila* (Emery et al. 1998; Stanewsky et al. 1998), whereas the mouse mCRYs act as light-independent core components of the circadian clock in mice (van der Horst et al. 1999). At about the same time, CRYs and phytochromes were shown to mediate light entrainment of the circadian clock in Arabidopsis (Somers et al. 1998). All CRYs discovered by that time showed regulatory activity of gene expression, without DNA photolyase activity. Subsequently, a third type of CRY, referred to as CRY-DASH (Drosophila, Arabidopsis, Synechocystis, Human) (Brudler et al. 2003) or CRY3 (Kleine et al. 2003), was discovered. It was found that CRY-DASH is a DNA-binding protein that affects gene expression (Brudler et al. 2003), and Arabidopsis CRY3 has a signal peptide that targets it into chloroplasts and mitochondria (Kleine et al. 2003). In contrast to the canonical CRYs that have no enzymatic activity repairing UV-damaged DNA, the DASH-type CRYs from bacteria and plants act as light-dependent ssDNA repairing DNA photolyases (Huang et al. 2006; Selby and Sancar 2006). The DASH-type cryptochromes lack an efficient flipping mechanism for repairing cyclobutane pyrimidine dimers within dsDNA, which explains why CRY-DASH cannot repair dsDNA (Pokorny et al. 2008). The function of DASH-type CRYs appears important to the genome stability of bacteria and organelles of eukaryotes because these smaller genomes may have relatively more abundant “melted” ssDNA regions resulting from rapid DNA replication and transcription. In the subsequent sections of this article, CRYs refer only to the canonical cryptochromes, especially Arabidopsis CRY1 and CRY2, that show no enzymatic activity in repairing cyclobutane pyrimidine dimers of DNA but function instead as signaling photoreceptors that regulate gene expression and photomorphogenesis.

Studies of the evolutionary history of photolyase/cryptochrome families indicate that ancient DNA photolyases may have duplicated multiple times during evolution, resulting in expansion of the photolyase/cryptochrome families and divergence of plant and animal CRYs more than 1 billion years ago (Lin and Todo 2005; Kim et al. 2014; Mei and Dvornyk 2015; Deppisch et al. 2022). This is consistent with the hypothesis that CRYs are the first sensory photoreceptors that evolved in plants (Han et al. 2019). It appears that CRYs in different eukaryotes may have arisen independently from different photolyases, which would explain the different modes of action of the different CRYs in different eukaryotes, despite their common role in regulating the circadian clock (Cashmore 2003; Sancar 2016). Up to now, neither canonical CRY nor CRY-DASH DNA photolyase has been found in Archaea (Deppisch et al. 2022). Thus CRYs might not have existed 2 billion years ago when eukaryotes diverged from Archaea (Doolittle 1997; Williams et al. 2013), making the evolutionary history of CRYs about 1 to 2 billion years old. Although the canonical CRYs, such as plant and mammalian CRY1 and CRY2, do not have the catalytic activity repairing cyclobutane pyrimidine dimers of DNA, Arabidopsis CRY1 and CRY2 have been recently reported to promote repairing of DNA double-strand breaks (DSBs) in response to blue light (Guo et al. 2023). Plant CRY1 and CRY2 undergo blue light–dependent interactions with ADA2b, a subunit of the highly conserved GCN5 acetyltransferase that is often recruited to DSBs to facilitate DNA repair. The CRY2-ADA2b interaction enhances the ADA2b interaction with SMC5/6 (Structural Maintenance of Chromosome 5/6) that repairs DSBs by homologous recombination (De Piccoli et al. 2006) and/or DNA loop extrusion mechanisms (Pradhan et al. 2023). Therefore, canonical cryptochrome may have evolved with not only new functions in the regulation of gene expression and the circadian clock by protein-protein interactions (see later) but also reinvented its ancestral function in DNA repair by blue light–dependent protein-protein interactions. Given the similar DSB repairing mechanisms in eukaryotes, it would be interesting to examine whether metazoan CRYs also possess similar functions promoting DNA repairing reactions in response to blue light.

### Signal transduction mechanisms of cryptochromes

**Figure 3.**
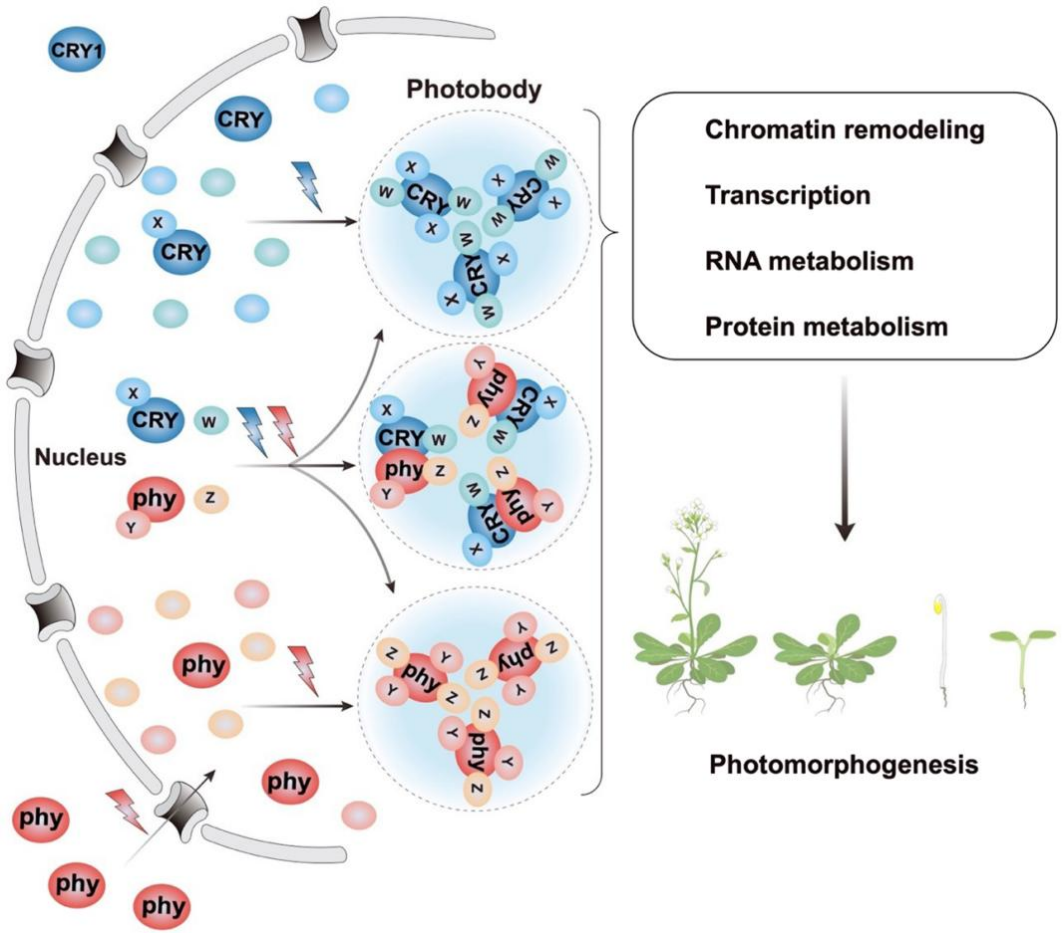
Light-triggered photobody formation by LLPS facilitates convergence of cryptochrome and phytochrome signaling pathways directly at the genome interface. The diagram depicts the nuclear importation of photoactivated phy and the condensation of both the photoactivated phytochromes and CRY by LLPS. This light-induced LLPS of the photoreceptors facilitates protein-protein interactions both between the phy and CRY molecules and with the numerous photoreceptor-bound signaling proteins, denoted W, X, Y, and Z. These multilateral interactions regulate processes that include chromatin remodeling, transcription, RNA metabolism (splicing, modification, degradation, etc), protein metabolism (translation, modification, degradation, etc), and photomorphogenesis.

The CRY signaling mechanisms were recently reviewed (Wang and Lin 2020; Ponnu and Hoecker 2022). Regulation of gene expression is the major cellular function of Arabidopsis CRYs, and the blue light–dependent formation or disintegration of CRY complexes composed of CRYs, CRY-signaling proteins, and CRY regulatory proteins is the major signal transduction mechanism of plant CRYs. For example, plant CRYs are known to mediate blue light promotion of anthocyanin biosynthesis (Sponga et al. 1986), and Arabidopsis CRY1 was shown to mediate blue light–induced expression of the mRNA encoding the key enzyme in anthocyanin biosynthesis, *CHS* (chalcone synthase) (Kubasek et al. 1992; Fuglevand et al. 1996). Soon after the Arabidopsis genome sequence was completed, Arabidopsis CRY1 and CRY2 were demonstrated to mediate blue light–regulated transcriptome changes (Ma et al. 2001; Folta et al. 2003; Ohgishi et al. 2004). Arabidopsis CRY1 (Cashmore et al. 1999) and CRY2 (Guo et al. 1999; Kleiner et al. 1999) are nuclear proteins. CRY2 is a “constitutive” nuclear protein that functions only in the nucleus (Yu et al. 2007a, 2007b), whereas CRY1 exists and functions in both the nucleus and cytoplasm (Fig. 3) (Wu and Spalding 2007). It is now clear plant CRYs interact with various gene expression regulators, including E3 ubiquitin ligases, transcription factors and co-factors, protein kinases, chromatin remodelers, splicing factors, RNA modifying enzymes, and phytochromes to regulate proteolysis, transcription, chromatin remodeling, mRNA splicing, mRNA modification, and translation, in response to blue light. For example, results of a recent multiple omics analysis indicates that, in the Arabidopsis *cry1cry2* mutant seedlings grown under continuous blue light, at least 7,400 genes showed significant changes of mRNA abundance in comparison to the wild type (Jiang et al. 2023a, 2003b), confirming that CRYs regulate mRNA expression of approximately one-quarter of the transcriptome (Ma et al. 2001). It is the light-dependent changes in association of the CRY protein complexes that cause the photoresponsive changes in gene expression and eventually photomorphogenic changes of plants (Wang et al. 2018a, 2018b; Wang and Lin 2020; Ponnu and Hoecker 2022).

Regarding proteolysis, similarly to that described for the phys above, the CRY-COP1/SPA-Transcription factor (TF) axis was the first discovered, and probably most extensively studied, mechanism of plant CRY signaling (Yang et al. 2000, 2001; Wang et al. 2001). Current understanding (Han et al. 2020; Wang and Lin 2020; Ponnu and Hoecker 2022) indicates that CRYs interact with the E3 ubiquitin ligase complex CUL4^COP1/SPAs^ to inhibit its activity, resulting in accumulation of the transcription factors, such as HY5, CO, LAF1, HFR1, and BBX21 (Wei and Deng 1996; Lau and Deng 2012; Kim et al. 2017a, 2017b; Han et al. 2020). Because these transcription factors act to promote transcription of genes required for photomorphogenesis, the CRY-dependent inhibition of CUL4^COP1/SPAs^ activity promotes photoresponsive changes of the transcriptome and proteome driving photomorphogenic development of plants. Arabidopsis CRYs interact with both COP1 (Wang et al. 2001; Yang et al. 2001) and the SPA1/COP1 complex in a blue light–dependent manner, imposing light-dependent inhibition of COP1 activity in the dark (Lian et al. 2011; Liu et al. 2011; Holtkotte et al. 2017). SPA1 is one of a 4-member family of COP1-interacting proteins that positively regulate COP1 activity (Hoecker et al. 1998; Lau and Deng 2012; Hoecker 2017; Podolec and Ulm 2018; Han et al. 2020). CRY2 also interacts with SPA1 in a light-dependent manner to inhibit COP1 activity, but photoexcited CRY2 may interact with COP1 in either a SPA-dependent or a SPA-independent manner (Zuo et al. 2011; Holtkotte et al. 2017; Ponnu et al. 2019). COP1 interacts with the VP motif of its substrates to cause polyubiquitination and degradation of the COP1 substrates (Holm et al. 2001). The VP motif in the CCE domain of Arabidopsis CRY1 and CRY2 is required for physiological functions of the CRYs (Lau et al. 2019; Ponnu et al. 2019; Liu et al. 2020) in their action as competitive inhibitors that suppress the CUL4^COP1/SPAs^ activity (Lau et al. 2019; Ponnu et al. 2019).

In addition to interacting with the COP1/SPAs complex to regulate proteolysis, CRYs interact with at least 80 other CRY-interacting proteins to regulate other aspects of gene expression, including transcription, chromatin remodeling, DNA repair, RNA splicing, and RNA modifications. For example, photoexcited CRYs interact with the bHLH transcription factors, called CIBs (CRY-Interacting bHLHs), to regulate flowering time in Arabidopsis or leaf senescence in soybean (Liu et al. 2008, 2013; Meng et al. 2013). CRYs also interact with the phytochrome-signaling bHLH transcription factors PIF4 and PIF5 to regulate shade avoidance and thermoresponses (Ma et al. 2016; Pedmale et al. 2016; He et al. 2022). CRYs interact with the transcriptional repressors PRR9 and TCP22, among others, to regulate the circadian clock (He et al. 2022; Mo et al. 2022). CRYs interact with hormonal responsive transcription regulators, such as IAAs, ARF6, BZR1, BIM1, GID, DELLA, the protein kinase BIN2, and the E3 ubiquitin ligases SAINTs, to modulate auxin, brassinosteroid, gibberellin, and ethylene responses, respectively (Wang et al. 2018a, 2018b; Xu et al. 2018; He et al. 2019; Mao et al. 2020; Lee et al. 2021; Xu et al. 2021; Yan et al. 2021; Zhong et al. 2021).

CRYs interact with these CRY-signaling proteins via 2 different domains of CRYs. All CRYs are characterized by a highly conserved N-terminal domain, referred to as PHR for Photolyase Homologous Region (Lin and Todo 2005), or CNT for CRY N-Terminus (Sang et al. 2005). Plant CRYs also possess a less conserved C-terminal domain unrelated to DNA photolyase, which was originally referred to as CCT (Yang et al. 2000), but was later renamed CCE (Cryptochrome C-terminal Extension) (Yu et al. 2010), to avoid possible confusion caused by the same domain name (IntePro# IPR010402 or Pfam# PF06203) registered for the CCT-family proteins (CONSTANS, CO-LIKE, and TIMING OF CAB1), that also have important functions in light signal transduction (Strayer et al. 2000). PHR is the chromophore-binding domain of CRYs that facilitates their blue light–dependent photoreactivity. Several lines of evidence support a hypothesis that absorption of blue-light photons by FAD changes the conformation of CRYs, including interactions between the PHR and CCE domains, resulting in the “open” conformation of the molecules that alter their protein-protein interactions with the CRY-signaling proteins, thereby altering gene expression (Yang et al. 2000; Partch et al. 2005; Yu et al. 2007a, 2007b; Goett-Zink et al. 2021). Consistent with this model, some CRY-signaling proteins, such as ADA2a/2b, AGB1, ARF6/8, BEE2, BIC1, CIBs, CIS1, CO, HBI1, IAAs, PhyB, TCP2, etc, interact with the PHR domain of CRYs, whereas others, such as ARP6, BES1, BZR1, MTA, RGA, SINATs, TOE1/2, etc, interact with both the PHR domain and the CCE domains (see review by Qu et al. 2023). The light-dependent conformational changes of the 2 different domains of CRYs may explain the complex network of the CRY-signaling proteins. However, the above domain-based classification of the CRY-interacting proteins is based on the reported results that were each analyzed under different conditions, leaving the general mechanisms underlying the structural specificity between CRYs and CRY-interacting proteins unclear at present.

### Photoactivation and inactivation of cryptochromes

Like all receptor proteins, the CRY photoreceptors undergo an activation and inactivation cycle upon light absorption. According to our current understanding, CRYs are photoactivated and inactivated/degraded by the following reactions. First, photoexcited CRYs change conformation to oligomerize, forming the CRY homo-tetramer that is physiologically active. This photoresponsive reaction of CRYs is referred to as CRY photoactivation (Sang et al. 2005; Rosenfeldt et al. 2008; Wang et al. 2016; Ma et al. 2020a, 2020b; Shao et al. 2020; Palayam et al. 2021). Photoreduction of the FAD chromophore of plant CRYs (Lin et al. 1995) has been hypothesized to be important for CRY photoexcitation, and its role in the mechanism of CRY activity has been previously reviewed (Liu et al. 2010; Chaves et al. 2011; Ahmad 2016). However, how FAD photoreduction is associated with oligomerization-dependent activation of CRY proteins remains unclear. Second, the oligomerized CRYs transduce the light signal by modulating the CRY complexes with or without changing the affinity between CRYs and CRY-interacting proteins (see below). Third, photoexcited CRYs are inactivated by interacting with BICs (Blue-light Inhibitor of Cryptochromes) that directly inhibit CRY oligomerization (Wang et al. 2016; Ma et al. 2020a, 2020b) and with 4 protein kinases PPKs (PPK1-4) that phosphorylate CRYs at more than 20 serine and threonine residues to not only stimulate CRY activity but also promote CRY ubiquitination and degradation (Shalitin et al. 2002; Liu et al. 2017; Gao et al. 2022). Two distinct E3 ubiquitin ligases, Cul4^COP1/SPAs^ and Cul3^LRBs^, catalyze CRY polyubiquitination to promote CRY degradation by the 26S proteosome (Shalitin et al. 2002; Chen et al. 2021; Liu et al. 2022; Miao et al. 2022). Although CRY1 appears stable upon relatively short exposure to blue light, 2 recent reports showed that Arabidopsis CRY1 is phosphorylated, ubiquitinated, and degraded in response to prolonged exposure to blue light via the same mechanism as CRY2 (Liu et al. 2022; Miao et al. 2022). It is interesting that PPKs, COP1, and SPAs are required for both the CRY function and CRY degradation. Fourth, the oligomerized CRYs undergo spontaneous monomerization in darkness (Liu et al. 2020). Distinct from photo-inactivation of CRYs that requires the blue light–dependent CRY-BIC interaction, this light-independent CRY monomerization process presumably results from thermal relaxation, and other reactions, that reverse the CRY conformation, dissolving the CRY oligomers to inactivate the CRY photoreceptors in darkness (Liu et al. 2020).

### CRY photobodies and light-induced LLPS

Más and colleagues first showed that blue-light activation of Arabidopsis CRY2 induces its rapid coalescence into nuclear condensates (Más et al. 2000). These condensates were initially called CRY2 nuclear speckles (Más et al. 2000), or CRY2 nuclear bodies (Yu et al. 2009), and, more recently, CRY2 photobodies (Chen and Chory 2011; Zuo et al. 2012; Ozkan-Dagliyan et al. 2013). However, the molecular basis for this coalescence, as for the phy photobodies, remained enigmatic until 2021 when Lin and colleagues discovered that the Arabidopsis CRY2 molecule undergoes LLPS (Wang et al. 2021) to form these biomolecular condensates (Fig. 3).

Recently it has been shown that this CRY2 LLPS formation is dependent on blue light, the FAD chromophore, the Intrinsically Disordered Region (IDR) of the CCE domain, and phosphorylation of the photoreceptor (Wang et al. 2021; Mo et al. 2022; Jiang et al. 2023a, 2023b; Ma et al. 2023). It has also been reported that CRY2 LLPS determines its function in multiple light-dependent responses, including transcriptional regulation, post-transcriptional mRNA methylation, CRY-phy co-action (see below), light regulation of the circadian clock, and light regulation of chlorophyll homeostasis (Wang et al. 2021; Mo et al. 2022; Jiang et al. 2023a, 2023b). Therefore, in addition to the regulation of many blue light-dependent CRY-interacting proteins that change the binding affinity to CRYs in response to blue light, photoexcitation-induced oligomerization is also necessary for the CRY-dependent functions of the CRY-interacting proteins that do not change the binding affinity to CRYs in response to light. Instead, these CRY-interacting proteins depend on the blue light–induced LLPS for photoresponsiveness. For example, the blue light–induced CRY oligomerization and LLPS are necessary for the functions of CRY-interacting proteins, such as COP1 (Wang et al. 2001; Yang et al. 2001), MTA (Wang et al. 2021), TCP22 (Mo et al. 2022), MAC3A (Jiang et al. 2023a, 2023b), FIO1 (Jiang et al. 2023a, 2023b), and probably many more that do not appear to change the CRY-binding affinity in response to light. It has been proposed that the light-induced coalescence of the CRYs increases the local concentration of both the photoreceptor and CRY-interacting proteins within the condensates (CRY photobodies), thereby accelerating the ensuing biochemical reactions (Wang et al. 2021; Jiang et al. 2023a, 2023b).

The available data are consistent with the concept that the IDR of the CRY molecule is responsible for keeping CRYs in the liquid phase during the LLPS process, and that the PHR domain is involved in photoresponsiveness and intermolecular interactions of the CRY complexes (Wang et al. 2021). Indeed, both phy and CRY molecules have not only well-structured but also unstructured IDR-containing regions, suggesting that both photoreceptors utilize this bifurcated structure for their photoresponsive signaling function (Pardi and Nusinow 2021; Wang et al. 2021; Chen et al. 2022).

Although as mentioned above, phototropism was the first blue-light response studied in plants (Darwin 1881; Briggs and Huala 1999; Christie 2007), because of space constraints here, we have omitted detailed discussion of the phototropins, the second major blue-light photosensory receptors molecularly defined, as well as of ZTL/FKF/LKP2 and the UV-light receptors. The phototropins, which sense directional blue light and induce the phototropic plant responses, were discovered by Winslow Briggs and colleagues in the 1990s (Christie et al. 1998). Subsequent progress in this area can be found in a number of periodic reviews (Christie 2007; Briggs 2014; Morrow et al. 2018). The ZTL and related proteins have been reviewed by Christie et al. (2015), and UVR8 by Ulm and colleagues (Rizzini et al 2011; Podolec et al. 2021).

### phy-CRY signaling convergence

A variety of genetic and physiological studies over a number of years have indicated that the blue- and red-light photoreceptors function both synergistically and antagonistically in regulating responses to variations in the natural daylight spectrum (Casal 2000; Lin 2000; Folta and Spalding 2001; Usami et al. 2004; Imaizumi and Kay 2006; Nozue et al. 2007; Su et al. 2017). Following identification of the phy and CRY photoreceptor molecules, there were several reports of the physical interaction between various family members of the 2 molecules by co-immunoprecipitation in vitro [phyA and CRY1; (Ahmad et al. 1998)]; phyB and CRY2; (Más et al. 2000) and by co-localization of phyB and CRY2 in nuclear speckles within the cell [by fluorescence resonance energy transfer microscopy (Más et al. 2000)].

In addition, both CRY1 and CRY2 have been shown to physically interact with the phy-interacting PIF transcription factors in a blue light–dependent manner. For example, CRY1 binds to PIF3 and PIF4 (Ma et al. 2016), while CRY2 binds to PIF4 and PIF5 (Pedmale et al. 2016). CRY1 inhibits the transcriptional activation activity of PIF4, thereby reducing the hypocotyl elongation response to both blue light and high ambient temperature (Ma et al. 2016). On the other hand, the interactions of CRY2 with PIF4 and PIF5 result in promotion of hypocotyl elongation under low blue light, a condition mimicking canopy shade where blue light is filtered through the canopy (Pedmale et al. 2016).

As detailed above, phyA, phyB, CRY1, and CRY2 have all been shown to physically interact with the COP1-SPA complex (Yang et al. 2001; Ma et al. 2016; Pedmale et al. 2016). These interactions dissociate this complex (Lian et al. 2011; Liu et al. 2011; Zuo et al. 2011; Lu et al. 2015; Sheerin et al. 2015), disrupting its E3-ligase activity, thereby allowing accumulation of positively acting transcription factors (e.g. HY5, HFR1) that function in both the phy and CRY signaling pathways to promote photomorphogenesis under red/far-red and blue light conditions. Taken together, these studies suggest that the phys and CRYs interact physically both with each other and with common signaling partners to regulate plant development under the prevailing light conditions in nature.

The discovery that both the photoactivated CRY and phy receptors coalesce very rapidly to form photobodies in the nucleus by LLPS (Wang et al. 2021; Chen et al. 2022) poises the system to provide a new dimension of understanding of these light-signaling pathways (Fig. 3). Numerous cellular factors defined as interactors of one or both photoreceptor molecules have been shown to co-localize with them in photobodies. These factors are known to have a diversity of functional roles in the cell suggesting that the photobodies may function as component-sequestration sites, as well as highly condensed hubs of transcriptional activity, regulated protein modification and degradation, chromatin remodeling, regulated mRNA degradation, circadian clock regulation, and cross-talk with other signaling pathways, such as the plant hormones (Paik et al. 2017; Ronald and Davis 2019; Willige et al. 2021). Overall, the emerging picture is that the phy and CRY sensory photoreceptors nucleate the coalescence of large complexes inside shared photobodies, generating a highly concentrated, dynamic milieux of interacting partners, where numerous multivalent transactions are enhanced.

## Data Availability

There are no new data associated with this article.

## Dive Curated Terms

The following phenotypic, genotypic, and functional terms are of significance to the work described in this paper:

CRY2 Gramene: AT1G04400
CRY2 Araport: AT1G04400

PIF3 Gramene: AT1G09530
PIF3 Araport: AT1G09530

PIF4 Gramene: AT2G43010
PIF4 Araport: AT2G43010

PIF7 Gramene: AT5G61270
PIF7 Araport: AT5G61270

SPA1 Gramene: AT2G46340
SPA1 Araport: AT2G46340

CUL4 Gramene: AT5G46210
CUL4 Araport: AT5G46210

HFR1 Gramene: AT1G02340
HFR1 Araport: AT1G02340

BZR1 Gramene: AT1G75080
BZR1 Araport: AT1G75080

CRY3 Gramene: AT5G24850
CRY3 Araport: AT5G24850

EBF1 Gramene: AT2G25490
EBF1 Araport: AT2G25490

CRY1 Gramene: AT4G08920
CRY1 Araport: AT4G08920

PORA Gramene: AT5G54190
PORA Araport: AT5G54190

UVR8 Gramene: AT5G63860
UVR8 Araport: AT5G63860

ZTL Gramene: AT5G57360
ZTL Araport: AT5G57360

COP1 Gramene: AT2G32950
COP1 Araport: AT2G32950

PHYE Gramene: AT4G18130
PHYE Araport: AT4G18130

ARP6 Gramene: AT3G33520
ARP6 Araport: AT3G33520

BBX21 Gramene: At1g75540
BBX21 Araport: At1g75540

RGA Gramene: At2g01570
RGA Araport: At2g01570

pifQ Gramene: pif1 pif3 pif4 pif5
pifQ Araport: pif1 pif3 pif4 pif5

CHS Gramene: AT5G13930
CHS Araport: AT5G13930
